# Dynamic Metabolomic Landscape of the Murine Brain Across Acute to Chronic Stages of *Borrelia garinii* Infection

**DOI:** 10.3390/biom16101441

**Published:** 2026-10-03

**Authors:** Bintao Zhai, Qingli Niu, Hui Yang, Kai-Fei Guo, Yang Liu, Bibo Bao, Yaxin Zhou, Bing Li, Hong Yin, Jiyu Zhang

**Affiliations:** 1 Key Laboratory of Veterinary Pharmaceutical Development, Lanzhou Institute of Husbandry and Pharmaceutical Sciences, Chinese Academy of Agricultural Sciences, Ministry of Agriculture and Rural Affairs, Lanzhou 730050, China; zhaibintao@caas.cn (B.Z.); 1073324120290@st.gsau.edu.cn (B.B.); zhouyaxin@caas.cn (Y.Z.); libing@caas.cn (B.L.); 2 State Key Laboratory of Animal Disease Control and Prevention, College of Veterinary Medicine, Lanzhou University, Lanzhou Veterinary Research Institute, Chinese Academy of Agricultural Sciences, Lanzhou 730070, China; yinhong@caas.cn; 3 College of Medical, Qinghai University, Xining 810016, China; yanghui@qhu.edu.cn; 4 College of Chemistry, Biology and Environment, Yuxi Normal University, Yuxi 653100, China; gkf@yxnu.edu.cn; 5 College of Life Science, Ningxia University, Yinchuan 750021, China; liuyang@nxu.edu.cn

**Keywords:** *Borrelia garinii*, untargeted metabolomics, brain metabolome, diagnostic biomarkers

## Abstract

Lyme neuroborreliosis (LNB), caused by *Borrelia garinii*, often leads to persistent neurological deficits, yet early diagnosis remains insensitive and the temporal metabolic changes in the infected brain are largely unexplored. In this study, a murine model of *B. garinii* infection was established using 80 female BALB/c mice, and brain tissues were collected at acute 7 (days post-infection, DPI), mid- (15 and 21 DPI), and chronic (30 DPI) stages for untargeted gas chromatography–time-of-flight mass spectrometry (GC-TOF-MS) metabolomics. Thirty-one differential metabolites were identified, with downregulated metabolites predominating acutely and upregulated metabolites prevailing chronically. Twelve common metabolites, including creatine, lysine, and galactose derivatives, were consistently altered. KEGG analysis revealed disrupted amino acid metabolism, particularly valine, leucine, and isoleucine biosynthesis (ko00290), arginine and proline metabolism (ko00330), and aminoacyl-tRNA biosynthesis (ko00970). ROC analysis identified five candidate biomarkers, hydroxylamine, 2,3-dihydroxypyridine, galactose 1, galactose 2, and monostearin, with high diagnostic accuracy. This study provides a comprehensive temporal metabolomic landscape of the brain during *B. garinii* infection, revealing time-dependent metabolic reprogramming with sustained disruptions in amino acid and energy metabolism, and offering candidate biomarkers for improved diagnosis of central nervous system involvement in Lyme disease.

## 1. Introduction

Lyme disease (LD), which is caused by the spirochete *Borrelia burgdorferi* sensu lato, is the most prevalent vector-borne illness in the Northern Hemisphere, with an estimated 476,000 cases diagnosed annually in the United States alone and a substantial burden across Europe and Asia [1,2,3]. In China, *Borrelia garinii* (*B. garinii*) is a predominant genospecies, and seropositivity rates in high-risk populations have been reported to exceed 10% in northeastern and western provinces, highlighting the significant public health impact of this pathogen [4,5]. *B. garinii* is mainly distributed in the forest regions of northern China and is associated with neuroborreliosis, manifesting as lymphocytic meningitis, cranial neuritis, and other neurological symptoms [6,7]. Studies have shown that the highly expressed BBQ67 in the *B. garinii* genome affects its own genetic transformation [8], and the high abundance of BBQ67 expressed in brain tissue may be related to the tissue tropism of neuroborreliosis-associated *B. garinii* [7]. In addition, during infection, *B. garinii* can differentially express outer membrane proteins such as OspA, OspC, DbpA/B, and Erp to adapt to the transition between tick and mammalian hosts and to evade host immune clearance [7]. These findings suggest that the molecular and cellular characteristics of *B. garinii* and the potential mechanisms underlying its interaction with the host, particularly its tissue tropism, may be closely associated with factors such as differential expression of membrane proteins and complement inhibition capacity. While early infection often manifests as erythema migrans (EMs), the spirochete can disseminate to multiple organs, leading to diverse clinical manifestations including arthritis, carditis, and neurological involvement [9]. Among these, Lyme neuroborreliosis (LNB) represents a particularly serious complication, with *B. garinii* being strongly associated with neurological manifestations such as meningoradiculoneuritis and encephalitis [10,11,12]. Current diagnosis of LNB relies predominantly on indirect serological methods, particularly the demonstration of intrathecal anti-Borrelia antibody synthesis, which, together with compatible neurological symptoms and CSF pleocytosis, forms the basis of the European case definitions [10,13]. However, intrathecal antibody production may be absent early in infection, and antibodies persist for years after successful treatment, limiting their utility for assessing disease activity [14]. Treatment typically consists of 10–14 days of intravenous ceftriaxone or oral doxycycline, with most patients responding well [15,16]. Nevertheless, a subset of patients develops persistent symptoms despite appropriate therapy, and prolonged antibiotic treatment has consistently failed to provide benefit in randomized trials [17,18]. These diagnostic and therapeutic limitations underscore the need for a deeper understanding of the metabolic perturbations occurring within the CNS during *B. garinii* infection.

Despite advances in serological diagnostics, significant challenges remain in the accurate detection of LNB, particularly during early stages when antibody responses are often undetectable [1,19]. Current two-tier serologic testing exhibits limited sensitivity in acute infection, and cannot reliably distinguish active from resolved disease [20,21]. Furthermore, the pathophysiological mechanisms underlying neuroborreliosis remain incompletely understood, particularly regarding the metabolic disturbances that accompany central nervous system infection [22]. While metabolomic approaches have shown promise for identifying diagnostic biomarkers in early LD and post-treatment Lyme disease syndrome [23,24,25], comprehensive characterization of the brain metabolome during Borrelia infection remains limited. Existing studies have primarily analyzed serum and CSF, identifying alterations in eicosanoid, bile acid, sphingolipid, glycerophospholipid, and acylcarnitine pathways in early disease [22,26,27], as well as tryptophan-kynurenine axis dysregulation and mitochondrial energy stress markers in neuroborreliosis [28]. However, these systemic and cerebrospinal profiles may not fully capture the localized metabolic perturbations occurring within the brain parenchyma. Moreover, the temporal dynamics of cerebral metabolic changes from acute through chronic stages of infection have not been systematically characterized.

The brain is a critical target organ in disseminated LD, where infection can lead to neuroinflammation, mitochondrial dysfunction, and alterations in neurotransmitter metabolism [29,30]. Previous transcriptomic analyses have revealed sustained interferon-regulated gene expression in patients with early disseminated LD [31], while metabolomic studies have identified disruptions in amino acid and lipid metabolism in serum and urine [25,32,33]. However, these systemic profiles may not fully capture the localized metabolic perturbations occurring within the brain parenchyma. Moreover, the temporal dynamics of cerebral metabolic changes from acute through chronic stages of infection have not been systematically characterized.

In this study, we employed a comprehensive untargeted metabolomic approach to characterize the temporal metabolic landscape of brain tissue in a well-established murine model of *B. garinii* infection. By analyzing metabolic alterations at acute (7 days post-infection, DPI), mid (15 and 21 DPI), and chronic (30 DPI) stages, we aimed to achieve three objectives. The first was to characterize time-dependent metabolic perturbations in the infected brain. Building on this, we sought to elucidate the key metabolic pathways disrupted during neuroborreliosis. Finally, we aimed to evaluate potential metabolite biomarkers that could facilitate improved diagnosis of central nervous system involvement. The brain focused metabolomic investigation addresses a critical gap in our understanding of LNB pathogenesis and may provide a foundation for developing targeted diagnostic and therapeutic strategies.

## 2. Materials and Methods

### 2.1. Mouse Model and Sample Collection

The *B*. *garinii* SZ strain was maintained at the Lanzhou Veterinary Research Institute, Chinese Academy of Agricultural Sciences. The strain was cultured to logarithmic phase at 33 °C, harvested by centrifugation (16,100 *g*, 10 min, 4 °C), washed twice with PBS, and resuspended at a concentration of 10^6^/mL. A total of 80 specific-pathogen-free (SPF) female BALB/c mice (4 weeks old, approximately 22 g) were obtained from the Experimental Animal Center of Lanzhou Veterinary Research Institute (License No.: SCXK (Gan) 2020-0002). After one week of acclimatization, mice were randomly assigned to four control groups (CK, 7, 15, 21, and 30 DPI) and four experimental groups (7, 15, 21, and 30 DPI), with 10 mice per group. Experimental mice were subcutaneously inoculated with 0.2 μL of the SZ strain suspension (10^6^/mL), while control mice received an equal volume of sterile PBS. Animals were maintained at 22 ± 2 °C with a relative humidity of 50 ± 10% and a 12 h light/12 h dark cycle. Sterile standard rodent chow and autoclaved distilled water were provided ad libitum. Bedding, cages, and water bottles were changed regularly by trained animal care staff. All animal procedures were approved by the Animal Ethics Committee of Lanzhou Veterinary Research Institute (Permit No. LVRIAEC2024-06) and conducted in accordance with the Animal Ethics Procedures and Guidelines of the People’s Republic of China.

Brain tissues were collected at 7 DPI (acute infection), 15 and 21 DPI (mid-infection), and 30 DPI (chronic infection). From each mouse, 200 mg of brain tissue was sent to BIOTREE Company (Shanghai, China) for metabolomics analysis. Additional tissue samples were fixed in 4% paraformaldehyde for hematoxylin and eosin (H&E) staining at Servicebio Company (Wuhan, China), with the remainder stored at −80 °C. All animal by-products were disposed of in a harmless manner.

### 2.2. Metabolite Extraction and Derivatization

The metabolite extraction and derivatization procedures were optimized as follows. A 50 mg sample was transferred to a 2 mL tube, followed by the addition of 0.4 mL of a methanol-chloroform mixture (3:1, *v*/*v*) and 20 μL of L-2-chlorophenylalanine as an internal standard. The mixture was homogenized using a ball mill and then centrifuged at 16,100 *g* for 10 min at 4 °C to remove tissue debris and precipitated proteins. The resulting supernatant was collected and evaporated to dryness under vacuum prior to derivatization. Subsequently, two-step derivatization was performed. First, 80 μL of methoxyamine hydrochloride in pyridine (20 mg/mL) was added, and the mixture was incubated at 80 °C for 20 min. Then, 100 μL of BSTFA (containing 1% TMCS, *v*/*v*) was added, followed by incubation at 70 °C for 1 h. After the sample had cooled to room temperature, 10 μL of a fatty acid methyl ester (FAME) standard mixture was added to the quality control (QC) sample. The resulting solution was ready for GC-MS analysis. Detailed procedures were referenced from the literature [34].

### 2.3. Untargeted Metabolomics by Gas Chromatography and Quadrupole Time-of-Flight Mass Spectrum (GC-TOF-MS)

GC-TOF-MS analysis was performed using an Agilent 7890 (Version B.04.03, Agilent Technologies, Santa Clara, CA, USA) gas chromatograph coupled with a Pegasus HT time-of-flight mass spectrometer. Separation was achieved on a DB-5MS capillary column (30 m × 250 μm i.d., 0.25 μm film thickness). A 1 μL sample was injected in splitless mode with helium as the carrier gas. The oven temperature was programmed from 50 °C to 320 °C at 10 °C/min and held for 5 min. The ion source was operated in electron impact mode at −70 eV, and mass spectra were acquired in full-scan mode (*m*/*z* 85–600) at a rate of 20 spectra per second. Detailed procedures and parameters were as described in reference [35].

### 2.4. Rectification and Inspection of the Machine

To validate the quality of the sample and determine the optimal metabolite extraction and detection method for GC-TOF-MS analysis, a preliminary experiment was conducted. This experiment included an experimental group, comprising a randomly selected single sample, and a control group, which used a simple internal standard. In the formal experiment, the stability of the system was assessed by the retention time (R.T., measured in minutes) of the internal standard (L-2-chlorophenylalanine). There are two points that need special mention: (1) For the GC-Quad FiehnLib library, we have named derivatives by increasing numbers according to retention index, e.g., serine 1, serine 2 and serine 3 (for the derivatives with no one or two TMS-groups derivatizing the primary amino group). (2) The LECO/Fiehn metabolomics library was used to identify the compounds. It will give a similarity value for the compound identification accuracy. If the similarity is >700, we can think the metabolite identification is reliable. If the similarity is <200, the library will only use “analyte” for the compound name. If the similarity is between 200 and 700, the compound name is putative annotation. In our study, similarity >700 was selected as the compound (metabolite).

### 2.5. Data Processing and Analysis

Chroma TOF4.3X software (version 4.3X, LECO Corporation, St. Joseph, MI, USA) of LECO Corporation and LECO-Fiehn Rtx5 database were used for raw peaks exacting, the data baselines filtering and calibration of the baseline, peak alignment, deconvolution analysis, peak identification and integration of the peak area. The retention time index (RI) method was used in the peak identification, and the RI tolerance was 5000.

The method of experimental data analysis employs both univariate (fold change (FC), *t*-test (*p*-value), variable important for the projection (VIP)) and multivariate analyses. Multivariate analysis is further subdivided into unsupervised analysis (principal component analysis, PCA), and supervised analysis (orthogonal partial least squares discrimination analysis, OPLS-DA). The multivariate pattern recognition analysis on the normalized data was conducted using SIMCA software (Version 14, Umetrics AB, Umea, Sweden). PCA utilizes a LOG conversion + CTR format processing method for data scale conversion, allowing for automatic modeling and data analysis. The OPLS-DA model was also executed using the SIMCA software to optimize differences associated with the predictive component within the model. Initially, OPLS-DA employs the data scale conversion method of LOG conversion + UV formatting. The model’s quality was then evaluated through a 7-fold cross-validation, and the model’s validity was assessed based on R^2^Y (which represents the interpretability of the Y variable) and Q^2^ (which signifies the model’s predictability) obtained post cross-validation. Subsequently, the model’s validity was further verified by modifying the order of the categorical variable by randomly organizing the experiments multiple times (n = 200). This provided different random Q^2^ values, furthering the validity testing of the model.

Therefore, the screening conditions for differential metabolites in this experiment were VIP > 1, *p*-value < 0.05, FC > 2 or <0.5. The differential metabolites between joint groups were represented through a Venn diagram (http://www.ehbio.com/test/venn/#/, accessed on 1 February 2026), volcano plot and heatmap (https://www.bioinformatics.com.cn, accessed on 5 February 2026). These differential metabolites and the pathways they participate in were analyzed using the KEGG database (https://www.genome.jp/kegg/, accessed on 10 March 2026) and GO database (https://geneontology.org/, accessed 10 March 2026). The receiver operating characteristic (ROC, http://www.bioinformatics.com.cn/plot_basic_one_or_multi_ROC_curve_plot_106, accessed on 16 March 2026, AUC > 0.8) curve was utilized to evaluate the specificity of specific LD metabolites as biomarkers.

## 3. Results

### 3.1. Metabolite Identification and Ion Detection

**Figure 1:**
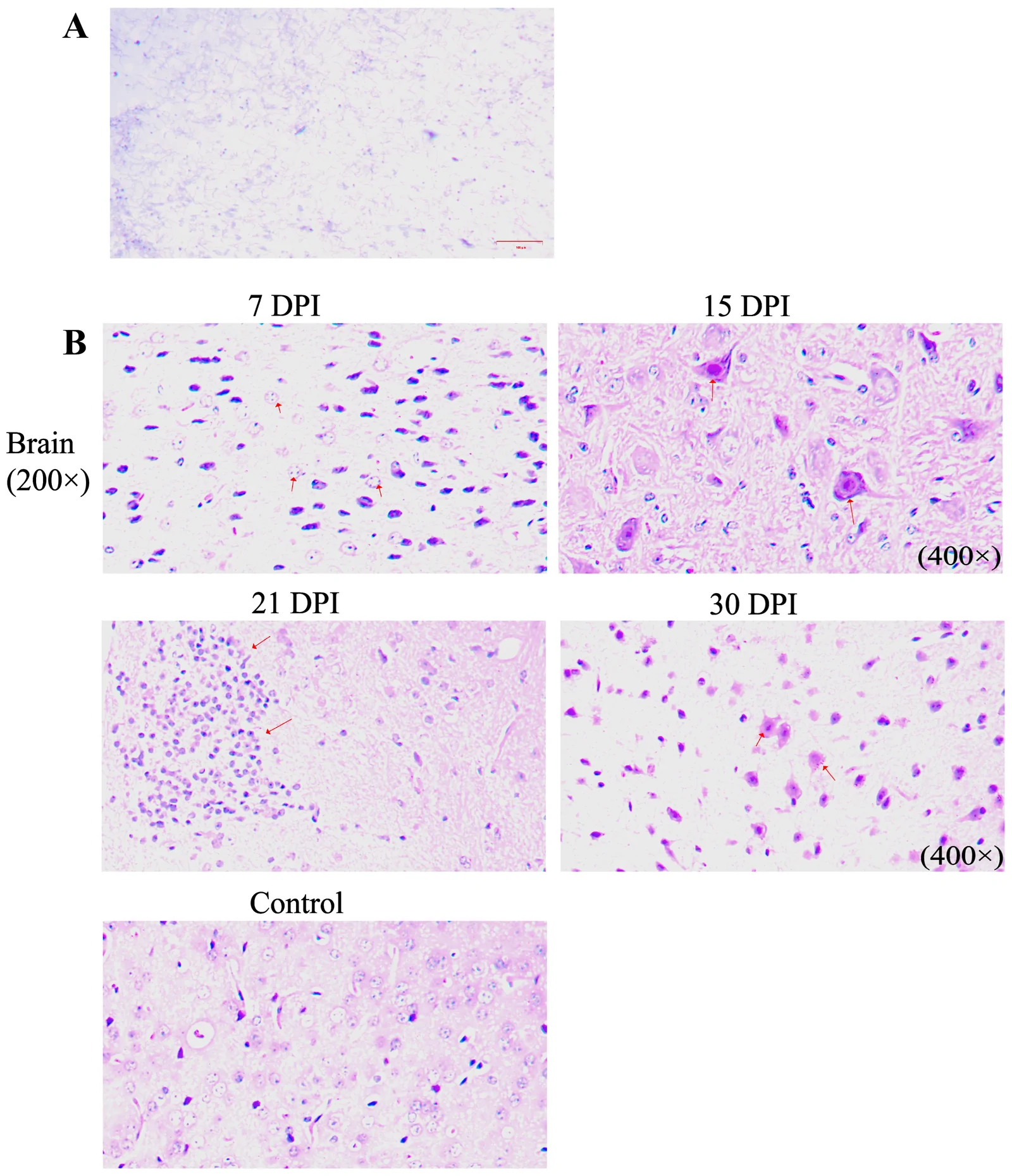
Validation of the murine brain infection model induced by *Borrelia garinii* SZ strain. Microscopic examination results of brain tissue infected with *Borrelia garinii* after isolation and culture (**A**). Histopathological analysis of brain from mice infected with the *B. garinii* SZ strain at 7, 15, 21, and 30 DPI (**B**). Brain from infected mice exhibited varying degrees of pathological lesions (indicated by red arrows), whereas no pathological changes were observed in the control group.

Successful infection with the *B. garinii* SZ strain was confirmed through multiple approaches. The mice brain tissue was isolated and cultured using BSK-H (Sigma, B3528, Shanghai, China). Under the microscope, spirillum bodies, exhibiting tremors and measuring between 10 and 15 Μm, were observable (Figure 1A). Moreover, histopathological analyses consistently verified spirochetal infection across all experimental mouse groups (Figure 1B). In addition, after 15 days of infection, we found that 8 of 40 infected mice (20%) had claudication or dragging in their hind limbs. These clinical observations, together with the microbiological and histopathological evidence described above, are consistent with the successful infection model reported in our previous study [36]. Culture and microscopy were performed on a subset of animals, whereas histopathology was performed on all groups, and that clinical signs were recorded daily for all animals throughout the study period.

Preliminary experiments confirmed excellent sample quality and optimal performance of the GC-TOF-MS analytical method and instrument platform (Figure S1), allowing the commencement of formal analyses. For metabolomic analysis, 80 samples were analyzed of brain. After preprocessing—including filtering, imputation of missing values, and standardization—a total of 510 peaks related to brain metabolites were retained for analysis (Additional File S1). Multivariate statistical analysis demonstrated clear data quality and group separations. Principal component analysis (PCA) was applied to the brain data across time points, and the distribution of the original data is shown in Figure S2. Orthogonal partial least squares-discriminant analysis (OPLS-DA) models, along with their corresponding permutation tests for the brain region and time point (Figure S3), demonstrated a clearer separation between groups, which facilitated the identification of variables driving classification. Model parameters indicated that all constructed models were stable, with good goodness of fit and strong predictive performance.

### 3.2. Screening of Differential Metabolites

**Figure 2:**
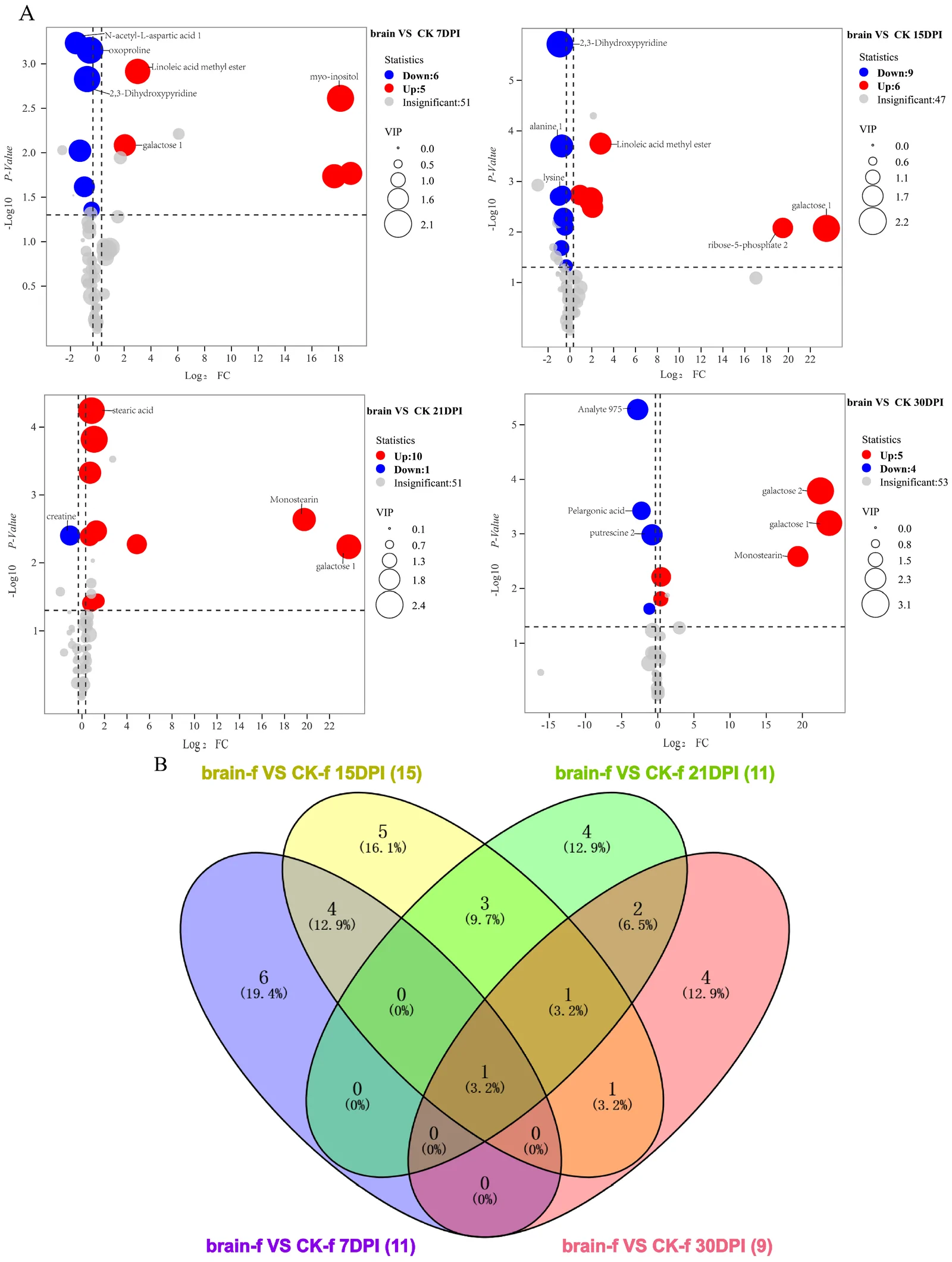
Volcano and Venn maps of differential metabolites across time points. Each figure represents the expression changes in metabolites in the brain at different time points (7, 15, 21, 30 DPI). The horizontal dashed line indicates the significance threshold *p* = 0.05, while the vertical dashed line indicates the fold change threshold |log2FC| = 1. Red and blue indicate upregulated and downregulated metabolites, and gray means that the difference is not significant, respectively. The top three significantly different metabolites that were up- and downregulated were labeled. A factor of difference (FC) > 2 or <0.5, VIP > 1, and *p*-value < 0.05 were used as the conditions for screening differential metabolites (**A**). The number of the cross section in the figure represents the common differential metabolites at each time point, and the percentage in brackets represents the ratio of these differential metabolites to the total differential metabolites (**B**).

**Figure 3:**
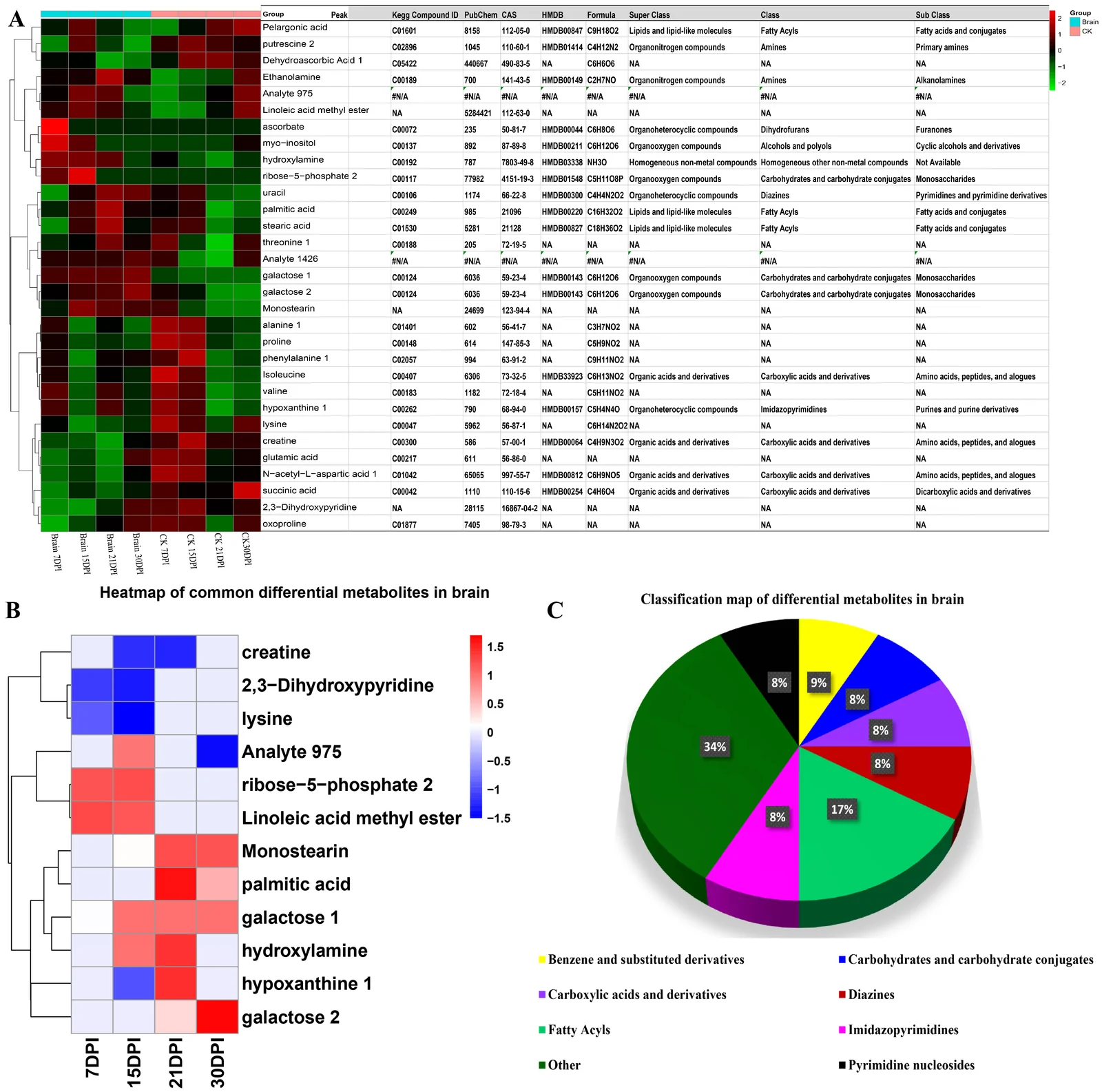
Heatmaps and classification of common differential metabolites. Cluster heatmaps display differential metabolites in the brain (**A**). Each row represents a single differential metabolite, and each column represents a sample at four time point. Color intensity reflects relative abundance, with green indicating lower and red indicating higher levels. Metabolite details are provided on the right side of the heatmap. (**B**) The left side is the heat map of the common differential metabolites of the brain at four time points (7, 15, 21, 30 DPI), (**C**) and the right side is the corresponding common differential metabolite classification map (one color represents a class of substances, and the percentage represents the proportion of this substance).

We identified 31 differential metabolites in the brain, with the top three significantly altered compounds highlighted in Figure 2 and detailed in Additional File S2. At 7 days post-infection (DPI), during the acute phase of infection, 11 metabolites were detected, of which 6 were downregulated and 5 upregulated. At the mid-stage of infection (15 DPI), 15 metabolites were identified, consisting of 9 downregulated and 6 upregulated. In the late stage (21–30 DPI), the number of detected metabolites gradually declined (11 at 21 DPI, and 9 at 30 DPI), with upregulated metabolites outnumbering downregulated ones (Figure 2A,B). Overall, the metabolite counts first increased and then decreased, with downregulated metabolites predominating in the early phase and upregulated ones becoming more prevalent in the later phase. To further illustrate the expression patterns and associated information of all differential metabolites, a heatmap and pie graph were presented in Figure 3A.

We further identified common differential metabolites for the brain at the four time points, the brain displayed 12 common metabolites, such as creatine, 2,3-Dihydroxypyridine, lysine, analyte 975, ribose-5-phosphate 2, linoleic acid methyl ester, monostearin, palmitic acid, galactose 1, hydroxylamine, hypoxanthine 1, galactose 2. Heatmap and classification analysis of these common differential metabolites (Figure 3B,C) revealed that the predominant categories were “others”, fatty acyls (17%), benzene and substituted derivatives (9%), carbohydrates and carbohydrate conjugates (8%), carboxylic acids and derivatives (8%).

### 3.3. Functional Analysis of Differential Metabolites

**Figure 4:**
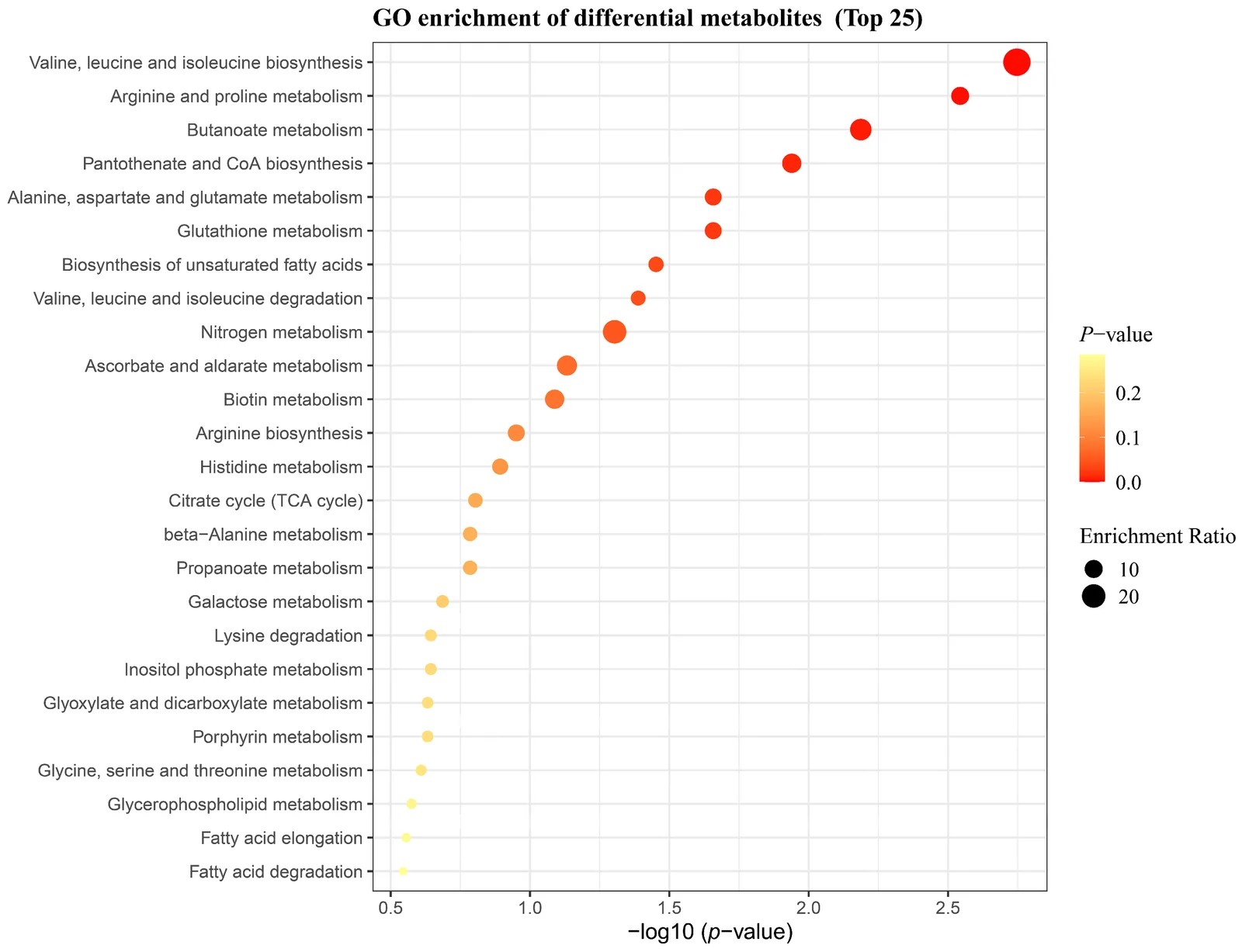
GO enrichment analysis of differential metabolites in brain. The smaller the *p*-value, the more significant the difference. The larger the enrichment ratio circle is, the more metabolites are involved, which represents the ratio of the enrichment degree of a candidate metabolite in a GO entry to the average enrichment degree in the entire reference background.

**Figure 5:**
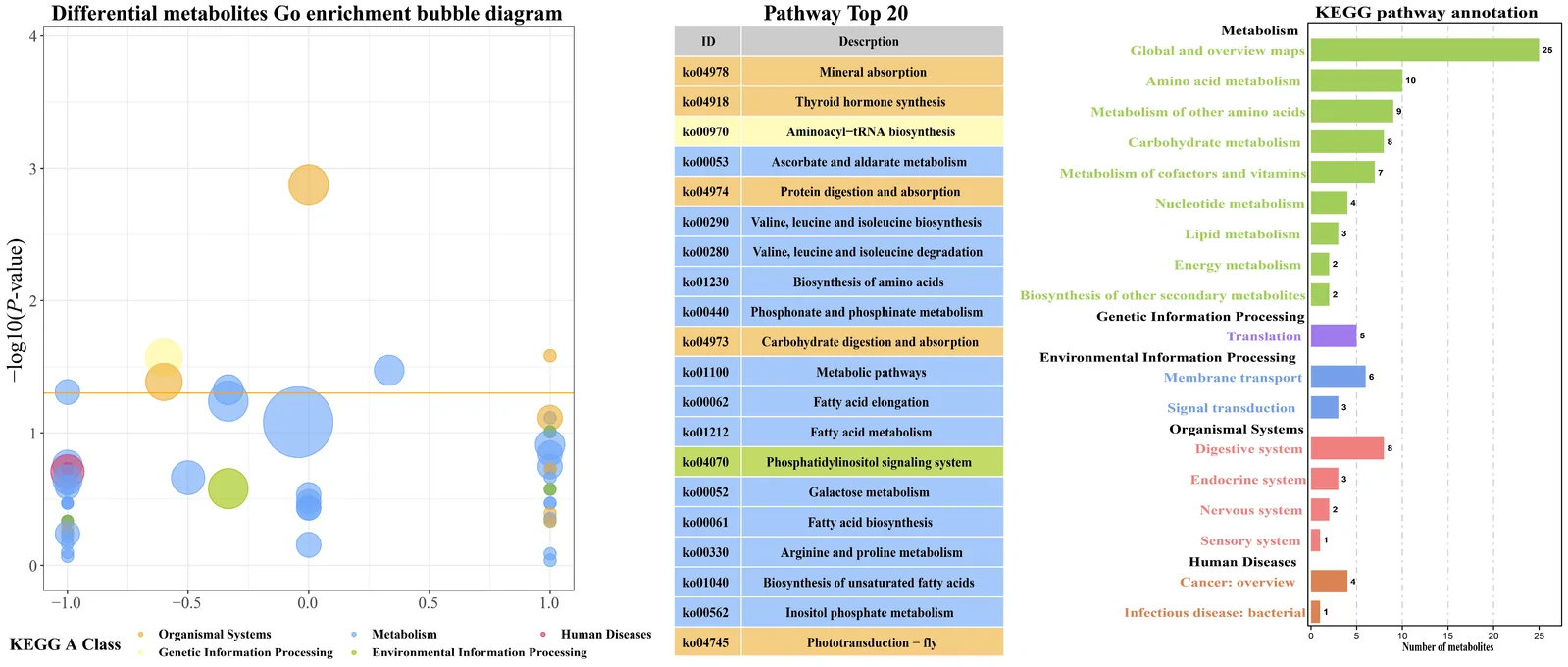
KEGG pathway enrichment analysis. The differential metabolites of the brain have three analysis diagrams. The first is the metabolite bubble diagram, the size of the bubble represents the quantity of enriched differential metabolites, while the color of the bubble corresponds to the primary classification in KEGG (divided into five or six categories: Organismal Systems, Genetic Information Processing, Metabolism, Environmental Information Processing, Human Diseases, Cellular Processes). The second is the top20 pathway name and its ID corresponding to the first map. The third is the KEGG pathway annotation map (including the primary and secondary classification of metabolite pathways), *x*-axis label represents the number of metabolites in the corresponding KEGG pathways in each KEGG subsystem. *y*-axis label represents main clusters of the KEGG pathways.

Enrichment analysis of significantly differential metabolites of the brain was performed in Figure 4. The two most significant enriched GO terms were “Valine, leucine and isoleucine biosynthesis” and “Arginine and proline metabolism”. We conducted KEGG annotation and enrichment analysis on the differential metabolites to assess their involvement in pathway regulation (up- or downregulation) and overall functional classification, as presented in Figure 5. Specific pathways such as “Global and overview maps” and “Amino acid metabolism” were prominently enriched. Based on enrichment classification, the majority of differential metabolites were associated with metabolic pathways, followed by organismal systems and human diseases pathway. The top 20 pathways were further analyzed, Aminoacyl-tRNA biosynthesis (ko00970), Ascorbate and aldarate metabolism (ko00053), Protein digestion and absorption (ko04974), Valine, leucine and isoleucine biosynthesis (ko00290), Valine, leucine and isoleucine degradation (ko00280), and Biosynthesis of amino acids (ko01230), were preferentially enriched.

**Figure 6:**
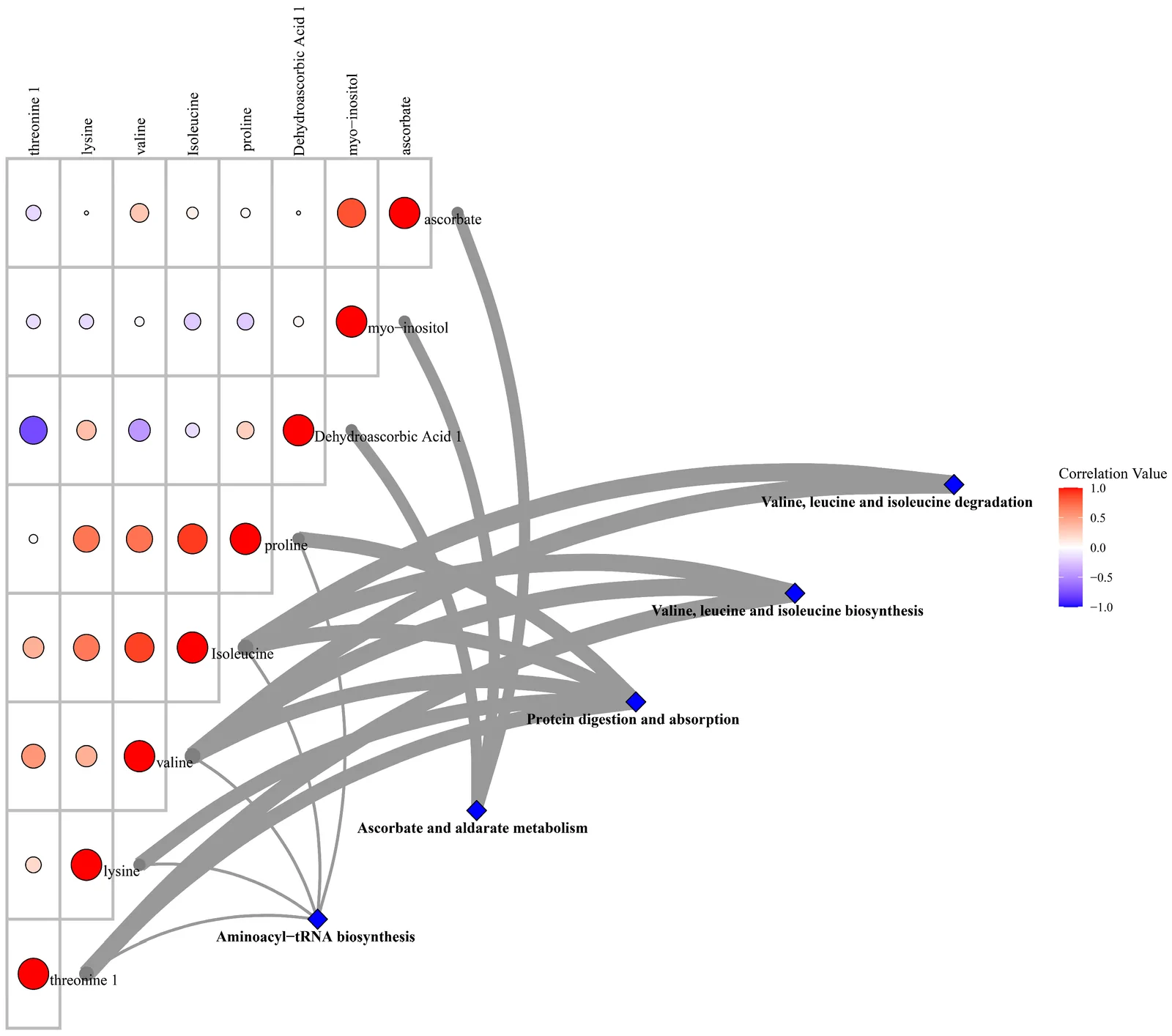
Correlation network of metabolites and pathways. The depth of the circle color and the size of the circle represent the intensity of the correlation between the metabolites. Red indicates a positive correlation, and blue indicates a negative correlation (correlation value). The line between the metabolite and the metabolic pathway indicates that the metabolite is involved in the pathway, and the thickness of the line indicates the *p*-value of the metabolite. Regarding, the *p*-value, the thicker the line is the smaller the value is.

Correlation networks between significantly altered metabolites and key KEGG pathways were further analyzed and visualized in Figure 6. The pathways displaying the highest correlation were “Protein digestion and absorption”, and “Aminoacyl-tRNA biosynthesis”, with key metabolites including myo-inositol, lysine, threonine 1.

To visualize regulation patterns of differential metabolites within key KEGG pathways, the up- or downregulation status of metabolites as summarized in Additional File S3. Metabolomic analyses revealed widespread disruptions in amino acid and central carbon metabolism in the brain following infection. Key pathways including arginine and proline metabolism (ko00330), alanine/aspartate/glutamate metabolism (ko00250), protein digestion and absorption (ko04974), and aminoacyl-tRNA biosynthesis (ko00970) exhibited coordinated downregulation of multiple metabolites (e.g., proline, lysine, valine, isoleucine, and succinic acid) at 7 and 15 DPI, with only threonine showing upregulation at 21 DPI. These findings highlight time-dependent and pathway-candidate metabolic perturbations indicative of sustained neuro metabolic dysregulation.

### 3.4. Searching for Potential Biomarkers

**Figure 7:**
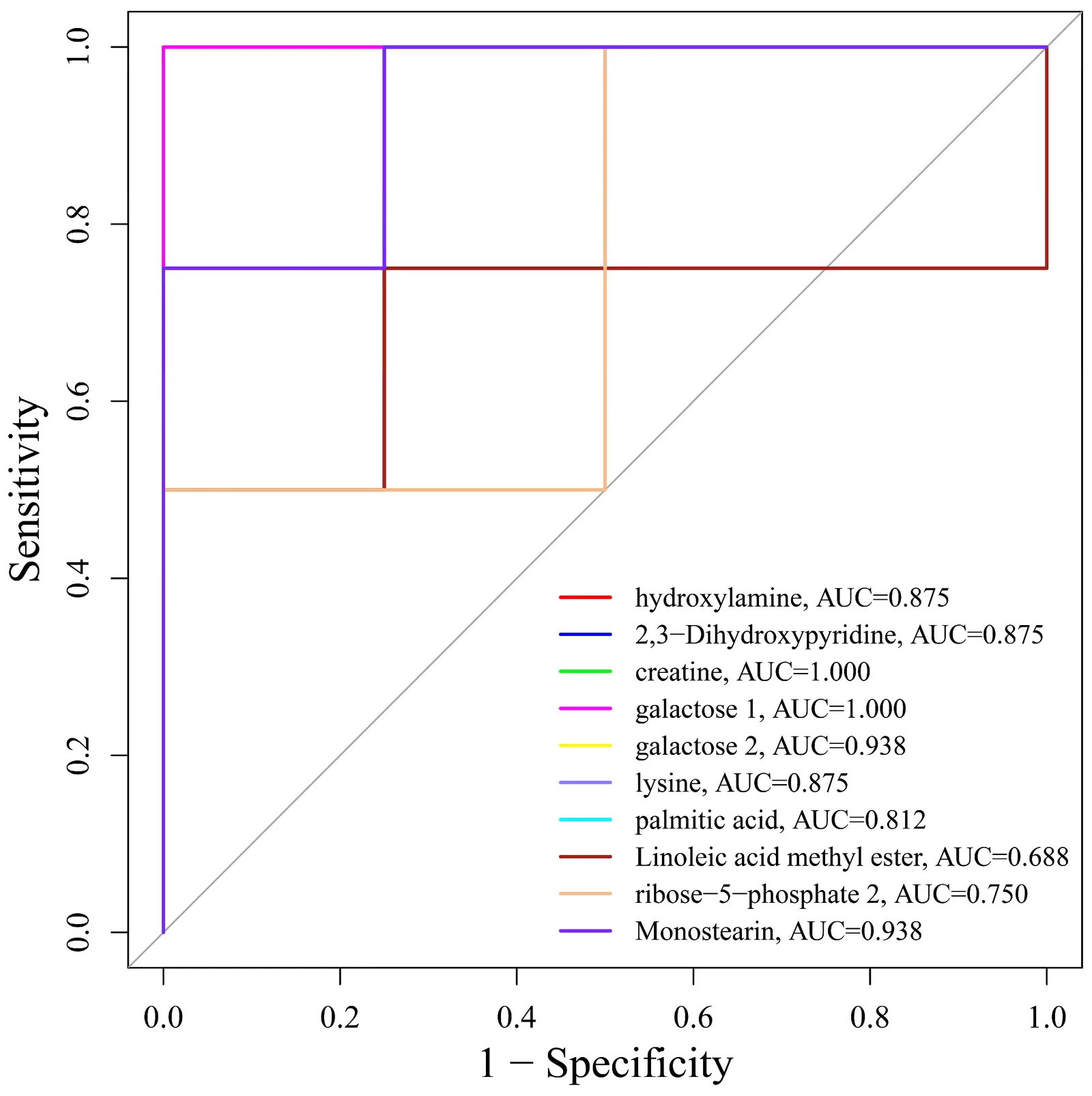
Receiver Operating Characteristic (ROC) curves of potential biomarkers. The *x*-axis represents the false positive rate, while the *y*-axis represents the true positive rate. The closer the area under the curve (AUC) is to 1.0, the higher the accuracy of the detection method or prediction. An AUC between 0.85 and 0.95 indicates a very good prediction effect, an AUC between 0.7 and 0.85 indicates an average effect, and an AUC of 0.5 or lower indicates the lowest accuracy, rendering the method or prediction ineffective. Different color lines represent different metabolites, and one region may overlap.

In the brain tissue, five potential biomarkers were successfully identified through the screening process, namely hydroxylamine, 2,3-dihydroxypyridine, galactose 1, galactose 2, and monostearin (Figure 7). These molecules were pinpointed as candidate biomarkers with potential diagnostic or research significance for relevant brain-related physiological or pathological studies, representing key molecular entities screened out from the brain tissue and laying a foundation for subsequent in-depth exploration of their biological functions and clinical application value in brain research.

## 4. Discussion

The present study applied an untargeted GC-TOF-MS-based metabolomics approach to characterize the temporal metabolic perturbations in the brain of *Borrelia garinii* infected mice, with a focus on identifying potential biomarkers and disrupted pathways. By integrating dynamic metabolomic profiles from acute (7 DPI), mid-(15, 21 DPI), and chronic (30 DPI) stages of infection, we identified 31 differential metabolites in the brain, revealing time-dependent shifts in host metabolism. Our findings are consistent with and extend prior observations on brain metabolic responses in Lyme disease (LD) [12,36], particularly highlighting the brain as a critical site of sustained metabolic dysregulation.

The brain exhibited a distinct metabolic profile with 31 differential metabolites identified. Temporal analysis revealed that downregulated metabolites predominated during acute infection (7 DPI), whereas upregulated metabolites became more prevalent at later stages (21–30 DPI), suggesting a shift from early metabolic suppression to a compensatory or maladaptive metabolic response during chronic infection. This pattern aligns with prior reports of *B. garinii* infection in mice, where bacterial load and host responses vary across brain and time points [37,38,39]. Indeed, Wu et al. demonstrated that *B. garinii* SZ strain preferentially colonizes the brain with bacterial loads peaking during the mid-to-late stages of infection [12]. This may explain the sustained metabolic alterations observed in the brain throughout the infection course.

Among the 12 common differential metabolites identified across all four time points in the brain, creatine, lysine, and galactose derivatives were prominent. Creatine, a key player in energy homeostasis, was downregulated in the brain at 15 and 21 DPI, consistent with its reduced levels observed in Alzheimer’s disease and other neurodegenerative conditions associated with mitochondrial dysfunction [40,41]. GC-TOF-MS detects only small molecules, with proteins removed during preparation. Given the sustained interferon-regulated gene expression reported in early disseminated Lyme disease, the amino acid and creatine depletion observed here may reflect downstream bioenergetic exhaustion from interferon-driven neuroinflammation rather than a direct pathogen effect. Integrated omics studies are needed to confirm this. In the context of LD, this may reflect impaired energy metabolism secondary to infection-induced neuroinflammation or direct pathogen-mediated disruption of cellular energetics. Lysine, another consistently altered metabolite, was downregulated at 7 and 15 DPI in the brain. Lysine is critical for protein synthesis and is a precursor for carnitine, which facilitates fatty acid oxidation [42,43]. Its downregulation may contribute to disruptions in energy metabolism and neurotransmission. In line with this, our pathway analysis identified significant enrichment of Aminoacyl-tRNA biosynthesis (ko00970) and Protein digestion and absorption (ko04974), with lysine and threonine emerging as key nodes in these pathways. These findings suggest that *B. garinii* infection may impair protein synthesis and amino acid availability in the brain, a phenomenon previously linked to the pathogen’s auxotrophy for several amino acids [44,45]. The identification of common metabolites in the brain, such as lysine and creatine, suggests shared host responses to *B. garinii* infection, while metabolites that were altered only at certain time points may reflect localized pathophysiological processes within the brain. These brain differential metabolites may be exploited for targeted diagnostic and therapeutic strategies.

KEGG enrichment analysis revealed that the most significantly impacted pathways in the brain included valine, leucine, and isoleucine biosynthesis (ko00290), arginine and proline metabolism (ko00330). The coordinated downregulation of valine, isoleucine, proline, and succinic acid at 7 and 15 DPI, with only threonine upregulated at 21 DPI, points to a dynamic and pathway metabolic response.

ROC curve analysis identified five potential biomarkers in the brain: hydroxylamine, 2,3-dihydroxypyridine, galactose 1, galactose 2, and monostearin, all with AUC > 0.8, indicating strong diagnostic potential. Among these, galactose derivatives have been previously associated with host glycosylation changes in acute LD, potentially affecting immunoglobulin function and immune evasion [46,47]. Hydroxylamine and 2,3-dihydroxypyridine are less commonly reported in LD but have been implicated in oxidative stress and neuroinflammatory processes in other contexts [48]. Monostearin, a glycerolipid, may reflect disruptions in lipid metabolism, which have been linked to Borrelia infection through altered lipase activity and membrane remodeling [49,50]. These biomarkers, though novel in the context of LD, align with the broader understanding of metabolic disruptions in infectious and inflammatory diseases. Their organ-specific nature underscores the utility of the brain metabolomic profiling for identifying diagnostic candidates tailored to particular manifestations of LD, such as neuroborreliosis. This study focuses on the brain as a primary target in Lyme neuroborreliosis and reveals that *B. garinii* infection drives substantial, time-dependent metabolic reprogramming within the central nervous system. A total of 31 differential metabolites were identified in the brain, with a clear temporal shift from predominantly downregulated metabolites in the acute phase to upregulated ones in the chronic stage. Persistent disruptions in amino acid and energy metabolism particularly involving creatine, lysine, and branched chain amino acids underscore the vulnerability of brain candidate biomarkers to infection-induced metabolic stress. These findings provide a detailed cerebral metabolic landscape that may inform the pathogenesis of neurological manifestations and offer brain biomarker candidates for diagnosing central nervous system involvement in Lyme disease. However, the brain displayed a unique enrichment of pathways related to amino acid metabolism and protein synthesis, reinforcing its vulnerability to infection-induced metabolic stress.

## 5. Conclusions

In summary, this study provides a comprehensive metabolomic characterization of brain responses to *B. garinii* infection, revealing time-dependent alterations in amino acid and energy metabolism, and identifying several candidate biomarkers with high diagnostic potential. Our findings highlight the brain as a metabolically vulnerable organ in LD and suggest that disruptions in protein synthesis, and central carbon metabolism may underlie the neurological manifestations of the disease. Future studies should validate these biomarkers in larger cohorts and explore their mechanistic roles in LD pathogenesis.

## Data Availability

The datasets supporting the findings of this article are included within the paper. The metabolomics data have been deposited in the Mendeley Data database, where we also uploaded our data (https://data.mendeley.com/drafts/c82ghsrfgd, accessed on 2 September 2026).

## Supplementary Materials

The following supporting information can be downloaded at: https://www.mdpi.com/article/10.3390/biom16101441/s1, Figure S1: Quality control assessment by GC-TOF-MS. The total ion current (TIC) chromatogram of quality control (QC) samples from the experimental group and the characteristic ion current chromatogram of the control group (simple internal standard) were obtained using GC-TOF-MS. The internal standard used was L-2-chlorophenylalanine (CAS: 103616-89-3, Shanghai Hengbai Biotechnology Co., Ltd., Shanghai, China). The TIC plot displays the time point on the *x*-axis and the summed intensity of all ions in the mass spectrum of each time point on the *y*-axis, generating a continuous profile. The QC sample TIC overlay plot was used to assess instrument performance. A higher degree of overlap indicates greater instrument stability; Figure S2: Principal Component Analysis (PCA) scores plots. PCA scores plots are presented for brain across the different time points (7, 15, 21, and 30 DPI). Samples predominantly fall within the 95% confidence interval (Hotelling T2 ellipse). The *x*-axis represents the first principal component (PC1), and the *y*-axis represents the second principal component (PC2); Figure S3: OPLS-DA model validation. Orthogonal Partial Least Squares-Discriminant Analysis (OPLS-DA) score plots for brain and the corresponding permutation test plots are provided. The *x*-axis represents the score for the first predictive component (t[1]P), and the *y*-axis represents the score for the first orthogonal component (t[1]O). Model quality was assessed using R^2^Y (representing the model’s fit) and Q^2^ (representing the model’s predictive ability), with values closer to 1 indicating a more robust model. Permutation test results are displayed with green circles (R^2^) and blue squares (Q^2^). The green and blue lines represent the regression lines for R^2^ and Q^2^, respectively; Additional File S1: Sample information and initial peak identification. This file lists the 80 samples analyzed per organ, their corresponding QC samples, and the associated peak numbers. Retention time (RT) is expressed in minutes (min), representing the time from sample injection to the point of maximum component concentration post-column. ID refers to the data identifier within the import software. ‘Count’ indicates the number of metabolites matched. ‘PEAK’ denotes the qualitative name assigned to each substance, and ‘similarity’ reflects the matching score between the identified substance and the standard library database (maximum score 1000). A higher similarity score indicates more accurate identification. Compounds with a similarity score below 200 are designated as ‘analyte’; Additional File S2: List of differential metabolites. This file provides the screening list of differential metabolites. ‘PEAK’ and ‘similarity’ are defined. ‘MASS’ value corresponds to the mass-to-charge ratio (*m*/*z*) of characteristic ions. ‘MEANXX’ indicates the normalized average peak area for each group. ‘Std.dev’ denotes the standard deviation between groups. The VIP (Variable Importance in Projection) value reflects the contribution of each variable to group separation. The *p*-value is derived from Student’s *t*-test. The fold change value represents the ratio between group means; Additional File S3: KEGG pathway analysis of differential metabolites. Color-coded diagrams illustrate the involvement of differential metabolites in KEGG metabolic pathways. Metabolites showing significant differential expression are marked in red (upregulated) or blue (downregulated). Note that the same metabolite may appear in brain within a pathway diagram, potentially with differing expression patterns.

## Abbreviations

The following abbreviations are used in this manuscript:

LD	Lyme disease
EMs	erythema migrans
LNB	Lyme neuroborreliosis
DPI	days post-infection
SPF	specific-pathogen-free
H&E	hematoxylin and eosin
QC	quality control
GC-TOF-MS	gas chromatography and quadrupole time-of-flight mass spectrum
RI	retention time index
FC	fold change
VIP	variable important for the projection
PCA	principal component analysis
OPLS-DA	orthogonal partial least squares discrimination analysis
ROC	receiver operating characteristic
AUC	area under the curve
TIC	total ion current
