# Supplementary material for: Dynamic Metabolomic Landscape of the Murine Brain Across Acute to Chronic Stages of Borrelia garinii Infection
Source: Biomolecules. 2026 Oct 3;16(10):1441. doi: 10.3390/biom16101441 (PMC13702289; doi:10.3390/biom16101441)
Supplement: Supplementary file 1 [file biomolecules-16-01441-s001.zip › Additional File S3.pdf]

Additional File S3A

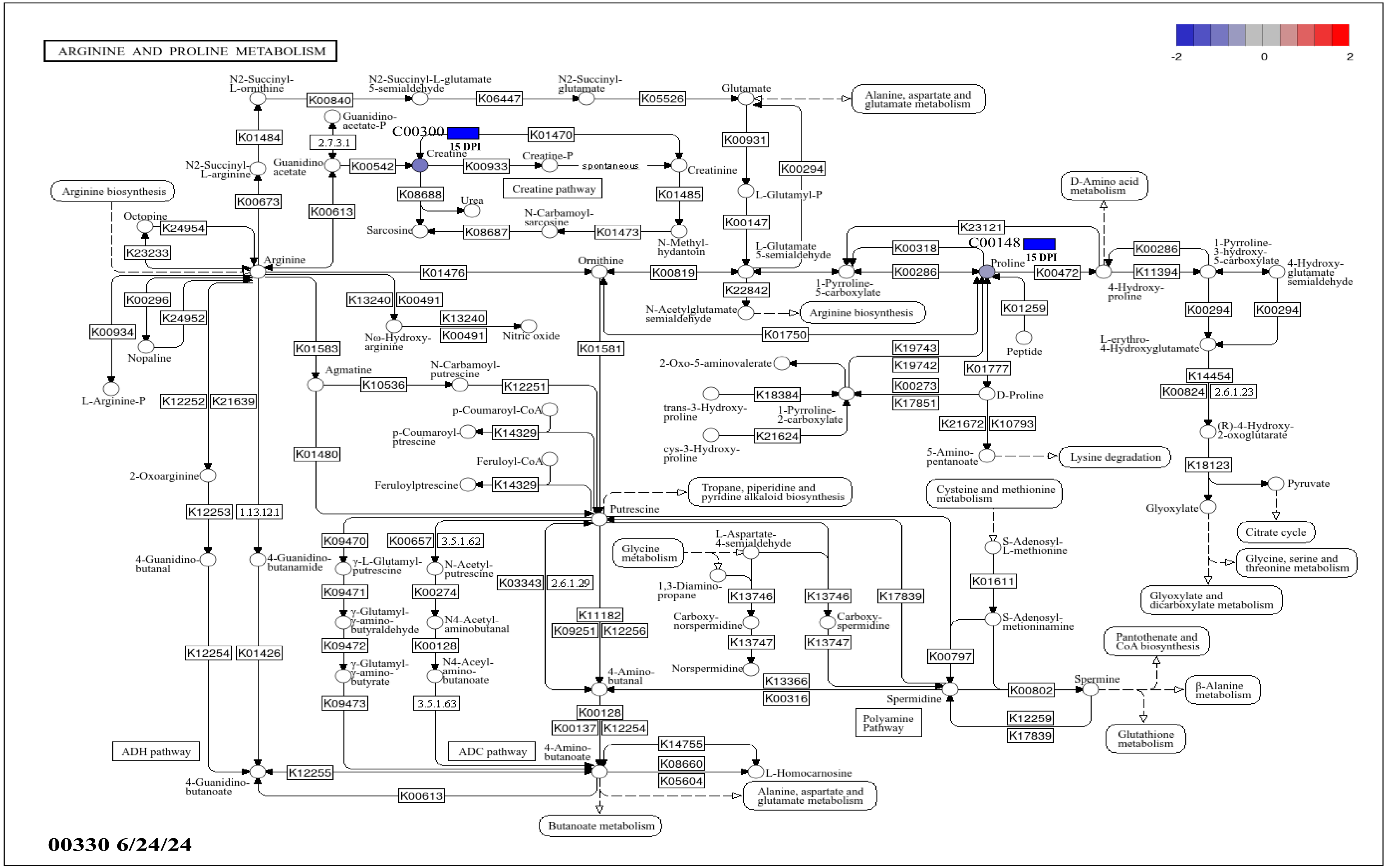

The arginine and proline metabolism pathway (ko00330) was enriched with 2 metabolites in the brain. Creatine (C00300) and Proline (C00148) were down-regulated at 15 DPI.

Additional File S3B

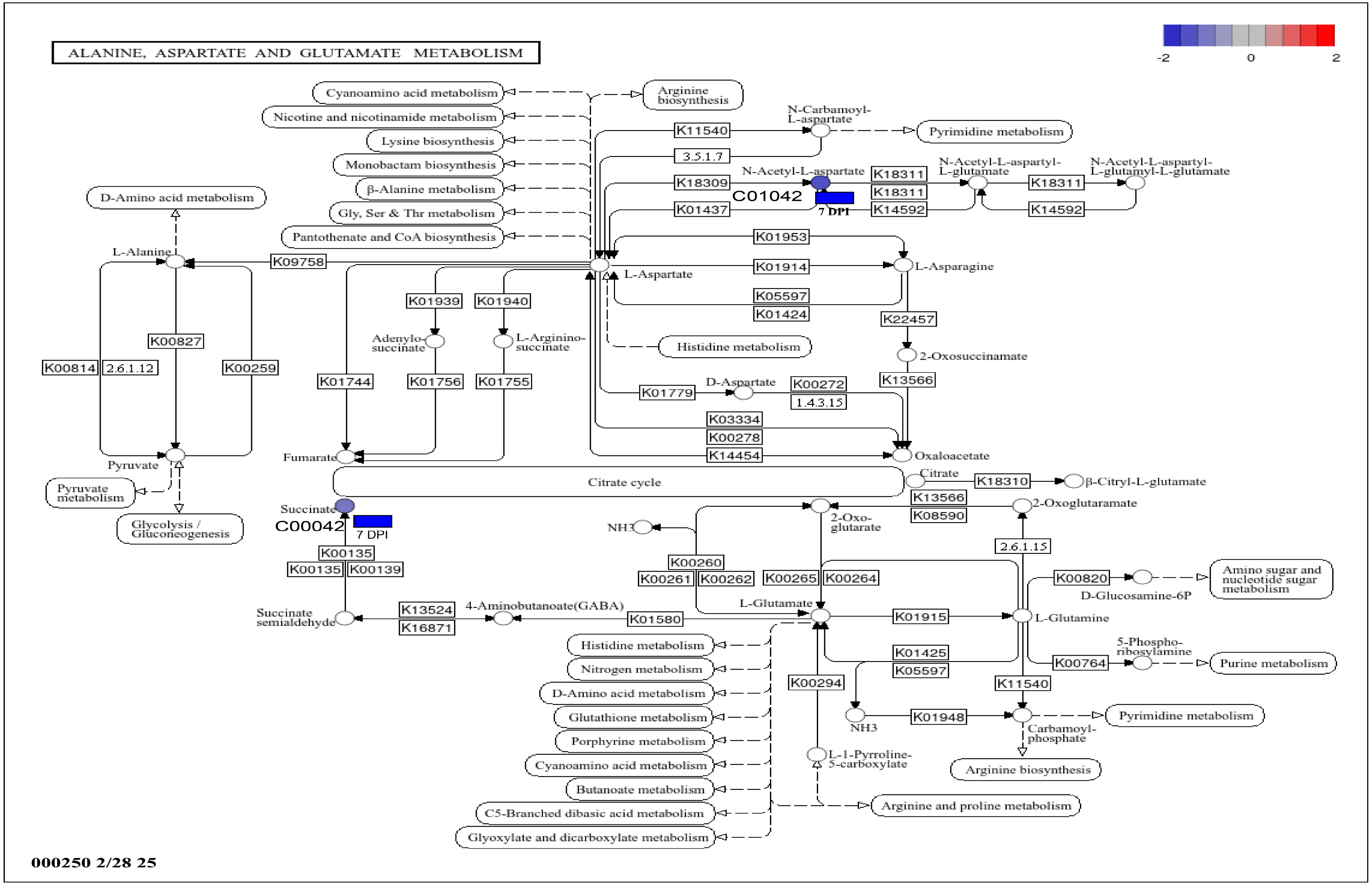

Alanine, aspartate and glutamate metabolism pathway (ko00250) screened 2 differential metabolites in the brain. Succinic acid (C00042) and N-acetyl-L-aspartic acid 1 (C01042) were down-regulated at 7 DPI.

Additional File S3C

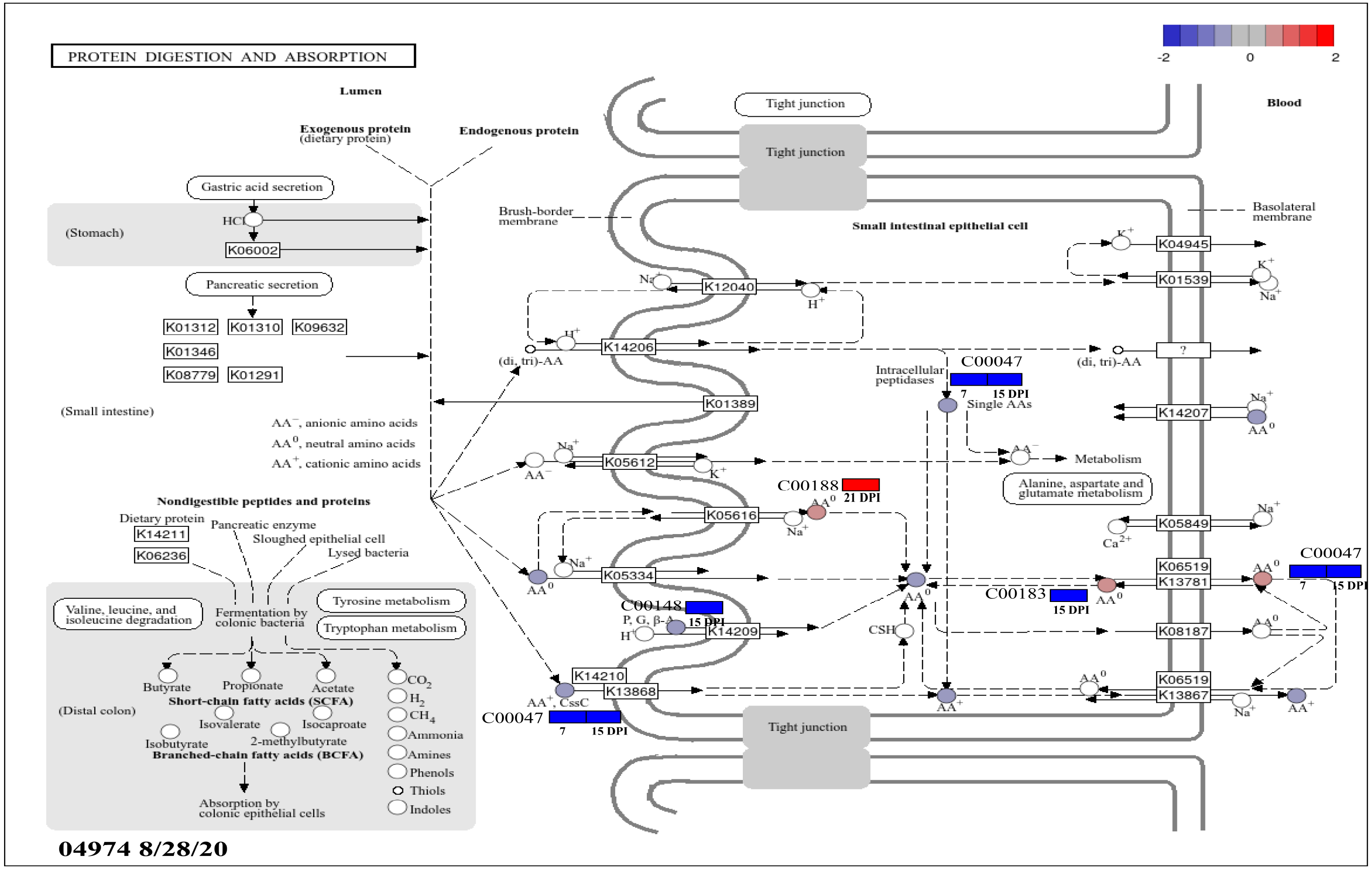

The protein digestion and absorption pathway (ko04974) screened 4 differential metabolites in the brain, with further subgroup variations (e.g., AA^0, AA^+, and single AAs) showing differential regulation at time points, underscoring the complexity of metabolic responses during infection. Proline (C00148) was down-regulated at 15 DPI. AA^+, CssC contains more than two substances (lysine-C00047, cystine-C00491) distributed. Lysine (C00047) was down-regulated at 7 and 15 DPI. AA^+ contains more than two substances (lysine-C00047, histidine 2-C00135) distributed. Lysine, proline, valine, and isoleucine at 15 DPI, lysine was down-regulated at 7 DPI.

Additional File S3D

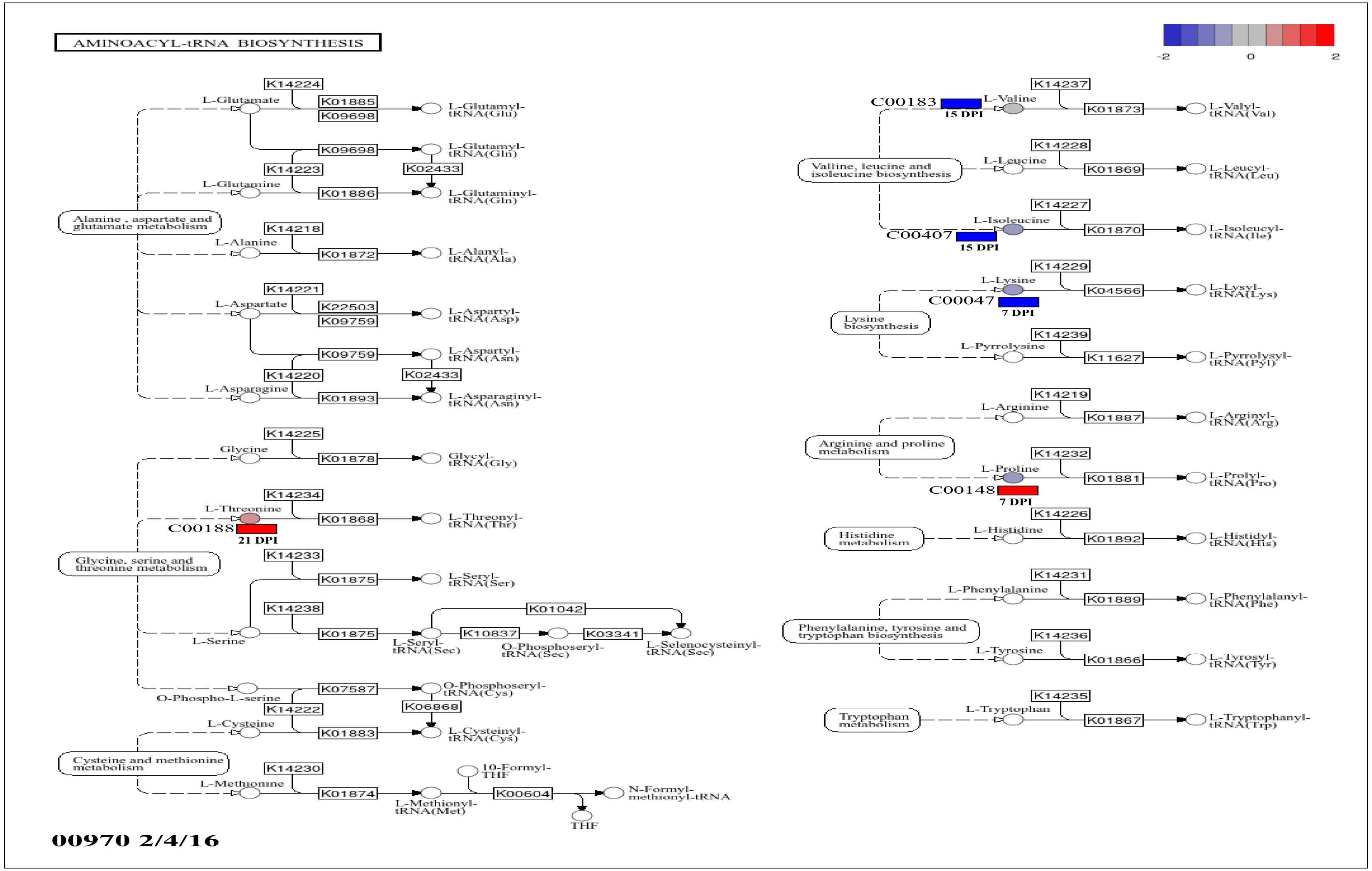

The aminoacyl−tRNA biosynthesis pathway (ko00970) involves 5 differential metabolites in the brain. Threonine 1 (C00188) was up-regulated at 21 DPI. Valine (C00183) was down-regulated at 15 DPI. Isoleucine (C00407) was down-regulated at 15 DPI. Lysine (C00047) was down-regulated at 7, 15 DPI. Proline (C00148) was down-regulated at 15 DPI.
